# Endocrine Maintenance Therapy in High-Grade Serous Ovarian Cancer: A Retrospective Off-Label Real-World Cohort Study

**DOI:** 10.3390/cancers17081301

**Published:** 2025-04-12

**Authors:** Franziska Geissler, Flurina Graf, Tibor A. Zwimpfer, Ruth S. Eller, Bich Doan Nguyen-Sträuli, Andreas Schötzau, Viola Heinzelmann-Schwarz, Ursula Gobrecht-Keller

**Affiliations:** 1 Gynaecological Cancer Centre, University Hospital Basel, 4031 Basel, Switzerland; franziska.geissler@usb.ch (F.G.); tiborandrea.zwimpfer@usb.ch (T.A.Z.); ruthstefanie.eller@usb.ch (R.S.E.); bichdoan.nguyen-straeuli@usb.ch (B.D.N.-S.); andreas.schoetzau@usb.ch (A.S.); ursula.gobrecht@usb.ch (U.G.-K.); 2 Ovarian Cancer Research, Department of Biomedicine, University of Basel, 4031 Basel, Switzerland; 3 Medical Faculty, University of Basel, 4031 Basel, Switzerland; flurina.graf@stud.unibas.ch

**Keywords:** high-grade serous ovarian cancer, endocrine therapy, maintenance therapy, aromatase inhibitor, letrozole

## Abstract

Background: Endocrine therapy is the standard-of-care maintenance treatment for estrogen receptor (ER)-positive breast cancers and is increasingly used in low-grade serous ovarian cancer. However, its therapeutic role in the early maintenance setting for ER-positive high-grade serous ovarian cancer (HGSC) remains undefined. Methods: A retrospective analysis was conducted on clinicopathological data from patients with newly diagnosed ER-positive HGSC following completion of adjuvant chemotherapy. Patients received maintenance therapy either with or without the aromatase inhibitor letrozole, in addition to standard maintenance care. ER expression levels and the administration of letrozole were analyzed, along with outcome measures for the entire cohort, with stratification based on residual disease status. Results: A total of 102 patients with newly diagnosed HGSC were included in the analysis, with 64 (62.7%) receiving letrozole and 38 (37.3%) not receiving letrozole. The median ER expression was 70%, with higher expression observed in the letrozole group compared to the no letrozole group (77.5% vs. 60%). No significant correlation was found between ER expression status and therapy response (*p* = 0.295 and *p* = 0.176, respectively). Letrozole therapy was well tolerated with no major adverse effects reported. In the overall cohort, maintenance letrozole therapy did not confer a significant improvement in progression-free survival (median 20.56 months vs. 29.34 months, *p* = 0.53) or overall survival (OS) (median 79.48 months vs. 46.85 months, *p* = 0.71) over a median follow-up duration of 23.5 months. However, among patients with no residual disease, maintenance letrozole therapy was associated with a statistically significant improvement in OS compared to those not receiving letrozole (median 114 months vs. 46.9 months, *p* = 0.006). Conclusions: Maintenance letrozole therapy appears to be a well-tolerated and potentially beneficial intervention in a subset of patients with ER-positive HGSC with no residual disease post-treatment. These findings highlight the need for further validation through prospective randomized trials to comprehensively assess the efficacy of endocrine therapy in this setting and its implications for patient quality of life.

## Simple Summary

Endocrine therapy is used for decades as a standard in the maintenance therapy in estrogen receptor positive breast cancer and now frequently discussed in low-grade serous ovarian cancers. For high-grade serous ovarian cancers only limited data in heavily pretreated patients is available. Thus, this study investigates endocrine therapy with the aromatase inhibitor letrozole alone or in combination with established targeted therapies in the maintenance after first line treatment with cytoreductive surgery and adjuvant chemotherapy in high-grade serous ovarian cancer patients. Our study demonstrates a good safety profile and thus feasibility for aromatase inhibitor letrozole in this setting. Moreover, Letrozole maintenance therapy shows a potential survival benefit in a selected group of high-grade serous ovarian cancer patients without residual disease after primary surgery. These findings highlight the potential of endocrine therapy as a well-tolerated option in high-grade serous ovarian cancers patients and the need for further validation through prospective randomized clinical trials to comprehensively assess its efficacy and its implications for patient quality of life.

## 1. Introduction

Ovarian cancer accounts for approximately 185,000 deaths worldwide annually, with high-grade serous ovarian cancer (HGSC) being the most common and lethal subtype, representing more than 70% of all epithelial ovarian cancer diagnoses [1]. The current standard of care for first-line treatment in HGSC involves cytoreductive surgery and six cycles of platinum and taxane-based chemotherapy [2]. While the majority of patients with HGSC initially respond well to chemotherapy, over 70% will relapse within three years, and ultimately develop resistance to treatment [2,3,4]. 

New treatment modalities, such as poly-ADP-ribosepolymerase (PARP) inhibitors and anti-angiogenic agents, have improved patient outcomes by extending progression-free survival (PFS) and overall survival (OS) [5,6,7,8]. However, these therapies are frequently associated with substantial toxicity, leading to treatment discontinuation (12–54%), dose reduction (28–70.9%), and dose interruption (20–79.5%) due to adverse effects [5,6,7,8,9]. This challenges underscore the necessity of identifying novel therapeutic approaches that increase PFS and OS without compromising quality of life.

The estrogen receptor (ER) is expressed in the majority of epithelial ovarian cancer cases, rendering it a potential target for endocrine therapy [10,11,12]. Following menopause, estrogen continues to be synthesized in peripheral tissues such as the liver, adipose tissue, brain, skin, and heart, despite the cessation of ovarian estrogen production [13]. Endocrine therapy has long been established as the standard maintenance treatment in ER-positive breast cancer [14,15].

In breast cancer, the American Society of Clinical Oncology (ASCO) and the College of American Pathologist defined ER positivity as nuclear staining in 1% or more of the tumor cells on immunohistochemistry [16]. In contrast, the definition of ER positivity in ovarian cancer is less consistent, with studies applying cut-offs ranging from >1% to 10% and 50% [17]. Endocrine agents including aromatase inhibitors (AIs), fulvestrant, gonadotropin-releasing hormone (GnRH) analogues, and tamoxifen have been primarily investigated in the recurrent setting, often in heavily pretreated patients [12,18]. The reported benefits of endocrine therapy in ovarian cancer have been inconsistent, and its potential synergy with other targeted therapies, such as PARP inhibitors or anti-vascular endothelial growth factor (VEGF) antibodies, remains uncertain.

While endocrine therapy is increasingly recognized as a viable option for low-grade serous ovarian cancer (LGSC), its role in the management of HGSC remains a subject of ongoing debate. We hypothesized that endocrine therapy may offer a well-tolerated and effective maintenance option, either alone or in combination with standard targeted therapies, in HGSC. The results of this investigation are the foundation of the ongoing ENGOT-ov54/Swiss-GO-2/MATAO trial [NCT04111978] [19].

## 2. Methods

### 2.1. Study Design, Study Population, and Study Size

**Figure 1:**
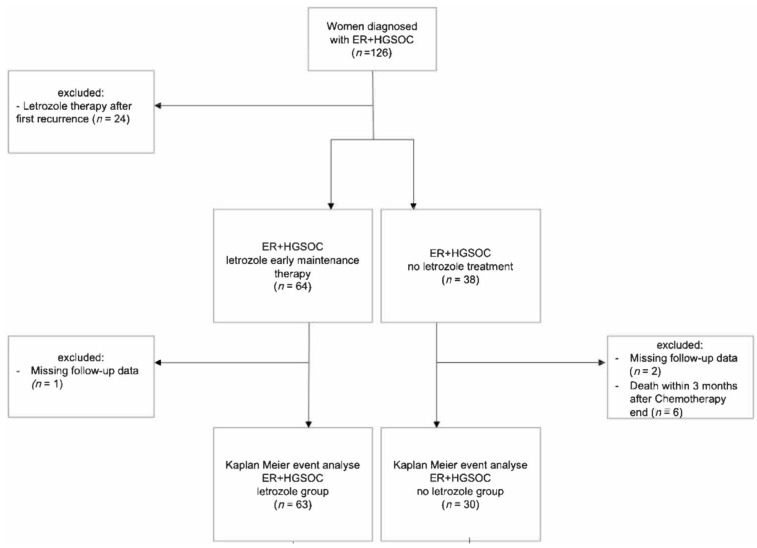
Flowchart of patient selection.

A single-center, retrospective cohort study was conducted at the Gynaecological Cancer Centre of the University Women’s Hospital Basel. We identified and analyzed the data of 102 patients with newly diagnosed ER-positive HGSC who received maintenance therapy with or without the AI letrozole (2.5 mg daily) between January 2013 and December 2021 (Figure 1). Letrozole was chosen as the AI due to its widespread use at our institution and its status as the most extensively studied AI in ovarian cancer management [20]. 

This study expands on a previously published pilot study involving 50 patients with ER-positive HGSC [21], incorporating data from 52 additional patients enrolled in subsequent years. 

The inclusion criteria for this study were (1) histologically confirmed high-grade serous ovarian, peritoneal, or fallopian tube cancer of all FIGO stages; (2) ER expression ≥ 1% confirmed by immunohistochemistry staining; and (3) standard care consisting of either primary debulking surgery followed by adjuvant chemotherapy or neoadjuvant chemotherapy with subsequent interval debulking surgery. The decision to administer letrozole was made collaboratively between patients and their physicians. Patients with missing follow-up data were excluded from the analysis, as were those who deceased within three months following completion of adjuvant chemotherapy. No exclusions were made based on FIGO stage or deviations from standard therapy due to clinical indications, reflecting the real-world and explorative nature of the study. During the study period, patients in Switzerland routinely received the anti-VEGF antibody bevacizumab in FIGO stages IIIC and IV or in cases with macroscopic residual disease (R) status, in accordance with Swiss-Medic approval. Additionally, since July 2019, Swiss-Medic has approved the use of the PARP inhibitor olaparib in maintenance therapy for patients with newly diagnosed epithelial ovarian cancer.

### 2.2. Variables, Data Sources, and Measurement

The primary endpoints of this study were PFS and OS. PFS was defined as the time from completion of first-line adjuvant chemotherapy until recurrence or death of any cause, with recurrence defined as symptomatic relapse confirmed by radiological examination. OS was defined as the time from diagnosis to death of any cause or last follow-up appointment. 

Various potential predictive and prognostic factors were evaluated, including ER expression, residual disease status, FIGO stage, age at diagnosis, and the use of letrozole therapy [17,18,19]. To ensure the accuracy of the analysis, potential confounding variables were identified and controlled for. These confounders included co-medication with established maintenance therapies, tumor burden, and variations in treatment duration and initiation timing. 

Clinicopathological data and disease progression information were collected by retrospective chart reviews. Side effects were documented during routine oncological follow-up visits every three months. For patients at increased risk for osteoporosis, bone density scans were conducted, and supplementation with Vitamin D, calcium, or denosumab was administered as required to mitigate potential bone loss. 

### 2.3. Quantitative Variables 

Patients were assigned to the letrozole group if they had received adjuvant letrozole maintenance therapy for at least three consecutive months. Those who declined or did not receive letrozole for any reason formed the control (no letrozole) group. Letrozole therapy could be initiated at any time after the initial diagnosis, and discontinuation was allowed at the patient’s discretion. Subgroup analysis was performed, stratifying patients with no residual disease (R0) and residual disease (R) after primary cytoreductive surgery.

### 2.4. Statistical Methods

Descriptive statistics were used to evaluate the clinical and demographic characteristics of the study cohort. Categorical data were presented as counts and frequencies, while metric or ordinal variables were expressed as medians (min, max). The Kruskal–Wallis test was used for metric and ordinal variables, and the chi-squared or Fisher’s exact test were used for categorical variables. A *p* < 0.05 was considered statistically significant. 

Time to event analyses were performed using the Kaplan–Meier method, and survival outcomes were compared using log-rank tests. Cox proportional hazard models were used to assess the potential influence of prognostic factors on patient outcomes. Missing data, concerning letrozole exposure, residual disease status, chemotherapy dates, and recurrence dates, primarily due to continuation of therapy at regional hospitals, were documented in a flowchart and excluded from the statistical analysis. All statistical analyses were conducted using R statistical software (version 4.1.3).

## 3. Results

### 3.1. Cohort Characteristics

The study cohort included 102 patients with ER-positive HGSC, including FIGO stages IC-IV. Among them, 8 patients (7.8%) were classified as FIGO stage I–II, while 94 patients (92.2%) had FIGO stage III-IV disease. The median age of the cohort was 67 years. All patients received standard-of-care treatment, including either primary debulking surgery followed by adjuvant chemotherapy (*n* = 72, 70.6%) or neoadjuvant chemotherapy with interval debulking surgery (*n* = 30, 29.4%). Platinum-resistant disease, defined as recurrence within six months of completing chemotherapy, was observed in eight patients (7.8%). A total of 64 patients (62.7%) received adjuvant maintenance therapy with letrozole, whereas 38 patients (37.3%) did not. The median follow-up time was 23.5 months. The demographic and clinicopathological baseline characteristics of the study population are summarized in Table 1, demonstrating an overall balanced distribution between the letrozole and no letrozole groups. Bevacizumab was administrated as part of adjuvant treatment in 38 patients (60.3%) in the letrozole group and 16 patients (43.2%) in the no letrozole group. A higher proportion of patients with a *BRCA1/2* mutation was observed in the letrozole group (28.6% vs. 10.5%). Additionally, residual disease was more prevalent in the letrozole group compared to the no letrozole group (55% vs. 34.3%). 

### 3.2. Safety Profile of Letrozole Treatment

No major adverse side effects of letrozole were observed in our cohort. Treatment interruption due to minor side effects (hot flushes, fatigue, arthralgia) was observed in eight patients (12.5%).

### 3.3. Effect of ER Expression on Letrozole’s Benefit

The median ER expression in the entire cohort was 70%, with a median of 77.5% in the letrozole group versus 60% in the no letrozole group. There was no significant interaction between ER expression and letrozole in relation to PFS (HR 1.02, CI 95% 0.987–1.045, *p* = 0.295) or OS (HR 1.05, CI 95% 0.979–1.124, *p* = 0.176).

### 3.4. Univariate Survival Analysis of Letrozole as Maintenance Therapy 

**Figure 2:**
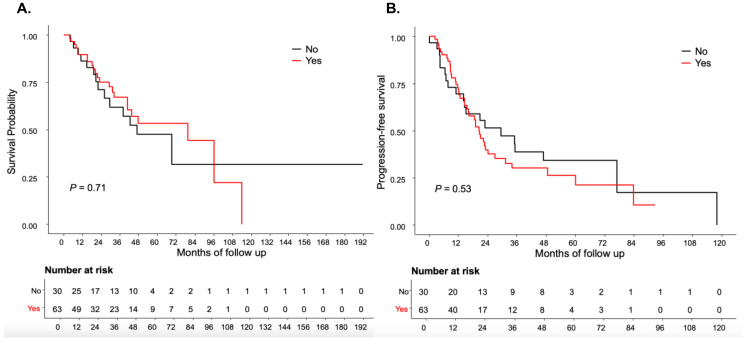
Kaplan–Meier curves of (**A**). overall survival and (**B**). progression-free survival of patients with early letrozole maintenance therapy compared to no letrozole maintenance therapy in high-grade serous ovarian cancer. OS = overall survival; PFS = progression-free survival.

No significant differences were observed in PFS and OS between patients who received letrozole and those who did not (*p* = 0.53 and *p* = 0.71) (Figure 2A,B). The median PFS was 20.56 months in the letrozole group versus 29.34 months in the no letrozole group, while the median OS was 79.48 months and 46.85 months, respectively.

### 3.5. Benefit of Letrozole Maintenance Therapy According to Residual Disease 

Residual disease status information was available for 95 patients (93.1%). Among these, 50 patients (52.6%) had R0 following primary cytoreductive surgery. In the R0 subgroup, 27 patients (54%) received letrozole, while 23 patients (46%) did not; within this subgroup, 18 patients (36%) and 15 patients (30%) received PARP inhibitors at some point in their treatment regimen. 

**Figure 3:**
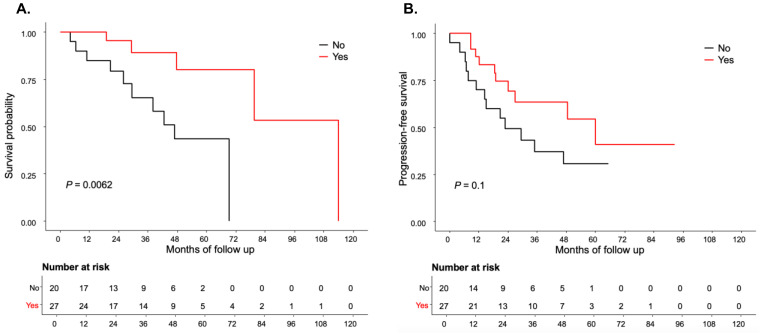
Kaplan–Meier curves of (**A**). overall survival and (**B**). progression-free survival of patients with early letrozole maintenance therapy compared to no letrozole maintenance therapy in high-grade serous ovarian cancer with no residual disease after primary cytoreductive surgery. OS = overall survival; PFS = progression-free survival.

Patients in the R0 letrozole subgroup demonstrated a statistically significant improvement in OS compared to the R0 no letrozole group (median 114 months vs. 56.85 months, *p* = 0.006) (Figure 3A). There was no statistical significance observed for PFS in the R0 letrozole group compared to the R0 no letrozole group (median 59.97 months vs. 22.79 months, *p* = 0.1) (Figure 3B). Conversely, in patients with R, no statistically significant association was found between adjuvant letrozole therapy and PFS or OS (*p* = 0.17 and 0.65, respectively) (Tables S1 and S2).

### 3.6. Benefit of Letrozole Maintenance Therapy After Multivariate Model Adjustment

The typical prognostic factors for ovarian cancer, including FIGO stage, age at diagnosis, residual disease, and primary tumor sites, were incorporated into the multivariate Cox regression model alongside treatment regimens with PARP inhibitors and bevacizumab. After adjusting for these variables, adjuvant therapy with letrozole remained statistically significantly associated with OS (HR 0.40, CI 95% 0.17–0.94, *p* = 0.035). 

**Figure 4:**
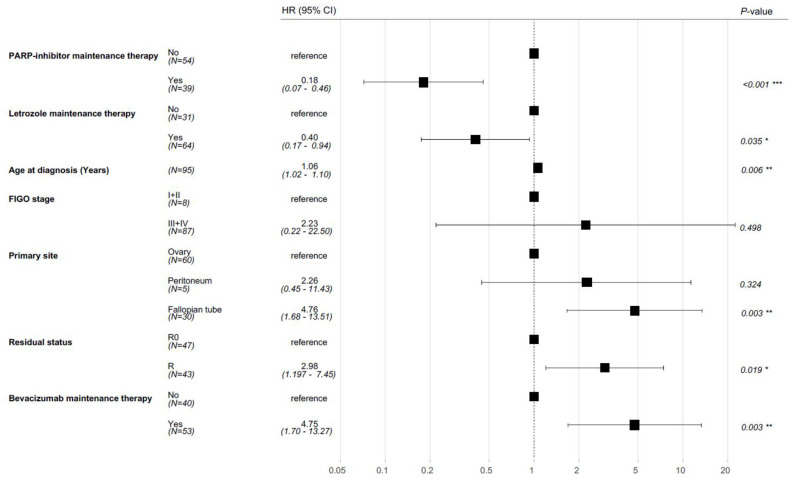
Forest plot of a multivariate Cox proportional hazard model of clinicopathological features predicting overall survival in patients with high-grade serous ovarian cancer. R = residual disease; R0 = no residual disease. (* *p* < 0.05, ** *p* < 0.01, *** *p* < 0.001).

In addition, PARP inhibitor therapy was associated with an OS benefit (HR 0.18, CI 95% 0.07–0.46 *p* < 0.001). As expected, residual disease (HR 2.98, CI 95% 1.97–7.45, *p* = 0.019) and adjuvant treatment with bevacizumab (HR 4.75, CI 95% 1.69–13.27, *p* = 0.003) were associated with poorer OS (Figure 4).

This analysis is exploratory in nature, and no formal sample size calculation was performed prior to the study. Based on an HR of 0.4 for OS (as observed in the multivariate model), a two-sided alpha of 5%, and a power of 80%, a total of 43 events would be required to detect a statistically significant difference. Our cohort included 37 events for OS, indicating that the study may be slightly underpowered to confirm the observed effect size with full statistical confidence.

## 4. Discussion

Due to the ability aromatase inhibitors for off-label use in Switzerland, we were able to generate a hypothesis regarding the application of letrozole, either alone or in combination with the anti-VEGF antibody bevacizumab or PARP inhibitors, as a maintenance therapy for patients with ER-positive HGSC. Our findings showed that HGSC exhibits a high median ER expression of approximately 70% and that endocrine therapy with letrozole is well tolerated, with a low discontinuation rate of 12.5% in our cohort. While the results from retrospective data cohorts are inherently limited by their non-randomized nature, our results suggest that patients with R0 after primary cytoreductive surgery may experience a survival benefit from adjuvant letrozole therapy. 

While endocrine therapy is heavily discussed in LGSC, this study represents the first real-world dataset analyzing aromatase inhibitor use in the adjuvant setting alongside routine targeted therapies in HGSC. Despite clear evidence that each ovarian cancer subtype has distinct molecular profiles and ER expression patterns [22], endocrine therapy has primarily been investigated in small Phase I and II trials involving heavily pretreated patients and mixed histological subtypes [12,18]. A meta-analysis of endocrine therapy in patients with recurrent epithelial ovarian cancer described a clinical benefit of 41%, highlighting the existence of a responsive subgroup [12]. However, the frequency of hormone receptor expression in HGSC remains variably reported and is often omitted from existing studies. In agreement with findings from the Ovarian Tumor Tissue Analysis (OTTA) consortium study [22], our cohort demonstrated a high median ER expression rate of 70%, consistent with prior analyses of treatment-naive and relapsed HGSC samples [23]. 

Beyond HGSC, ER expression has also been associated with improved disease-specific survival in endometrioid ovarian cancer [22]. This suggests that, in addition to HGSC and LGSC, the endometrioid subtype may also benefit from endocrine-targeted approaches. Conversely, other histological types, such as mucinous and clear-cell carcinomas, appear to lack consistent associations between ER expression and clinical outcomes, indicating limited applicability of endocrine strategies in these subtypes [22]. 

In breast cancer, ER expression serves as a predictive marker for response to endocrine therapy [24,25]. However, its predictive value in ovarian cancer remains unclear. Despite ER expression levels ranging from 1 to 100% in our cohort, no correlation was observed between the ER expression level and letrozole benefit in regard to PFS and OS. These results are consistent with the PARAGON Phase II trial, which found no association between ER histoscores and clinical benefit rates [26]. In contrast, some studies have reported higher response rates in patients with elevated ER histoscores [11] or a proportional relationship between clinical benefit and ER expression [27].

Both bevacizumab [28,29] and PARP inhibitors [5,6,7,8,9,30] have demonstrated efficacy in adjuvant and maintenance treatment settings for HGSC in multiple clinical Phase III trials. However, these therapies are frequently associated with substantial toxicities, including fatigue, hypertension, and hematological, gastrointestinal, and renal complications, leading to treatment discontinuation in 12–54% of cases [5,6,7,8,9,30,31,32,33]. Additionally, control arms in past clinical trials differed from those in contemporary endocrine therapy studies, where new treatment approaches are compared against established maintenance therapies such as PARP inhibitors and bevacizumab. During our study period, bevacizumab was well established, whereas PARP inhibitors were still emerging as a standard of care [5,9,34], explaining the lack of combination treatment with bevacizumab and olaparib in our cohort and the limited administration of niraparib.

The adjuvant maintenance phase is a critical period where curative potential may be maximized, necessitating careful evaluation of treatments based on adverse effects and cost–benefit ratios. In line with previously reported results showing low toxicity in recurrent ovarian cancer [35] and extensive experience in breast cancer [36], our findings confirm a favorable safety profile for letrozole in HGSC. As an orally administered agent, letrozole also offers the advantage of ease of use, which may promote adherence and patient satisfaction [27,37]. The most notable adverse effects of prolonged letrozole (2.5 mg/day) exposure include bone resorption and increased risk of hypercholesterolemia [38]. However, these effects can be mitigated with bisphosphonates and cholesterol-lowering agents. Furthermore, aromatase inhibitors did not show increased cardiovascular events when compared with a placebo in the extended-adjuvant setting in breast cancer [39]. Given this favorable toxicity profile, letrozole may represent a particularly appealing option for patients who are unable to tolerate more aggressive therapeutic approaches due to age, comorbidities, or performance status. 

Nonetheless, the therapeutic efficacy of endocrine therapy may be tempered by its slower onset of action compared to chemotherapy, making it less ideal in the setting of rapidly progressive disease [18]. In addition, the potential for resistance development poses as challenge to long-term treatment success [40]. Despite these challenges, endocrine therapy is considered a cost-effective treatment option in ER-positive ovarian cancer [41]. In LGSC, maintenance letrozole was shown to yield an incremental cost-effectiveness ratio of USD 11.037 per quality-adjusted life year, far below commonly accepted thresholds. While cost-effectiveness data specific to HGSC remain limited, the relatively low acquisition cost of endocrine agents and their favorable safety profile support their consideration as a cost-effective strategy in selected patients with HGSC [37].

The primary strength of this study lies in the off-label use of aromatase inhibitors in the adjuvant setting, a unique aspect facilitated by the Swiss regulatory framework. Additionally, to our knowledge, this is the first real-world cohort of patients with HGSC receiving endocrine therapy in the adjuvant setting, whereas previous analyses have focused on heavily pretreated populations. However, this study is inherently limited by its modest sample size, and its retrospective, single-center design introduces inherent selection biases. ER expression varied among patients, and the initiation of letrozole therapy occurred at variable time points following adjuvant chemotherapy. Furthermore, a selection bias favoring letrozole administration in patients with greater tumor burden cannot be excluded. Additionally, a positive *BRCA* mutation status may confound survival outcomes, as patients with *BRCA*-mutated tumors generally exhibit enhanced platinum and PARP inhibitor sensitivity compared to non-carriers [42]. However, the study cohort demonstrated comparable use of maintenance therapies between groups, as further confirmed through the multivariate analysis. 

### Implications for Practice and Future Research

This study underscores the potential utility of endocrine therapy as well-tolerated maintenance option in HGSC, particularly valuable for geriatric or medically frail patients. While limited by its retrospective design, our findings provide an essential foundation for ongoing and future prospective clinical trials evaluating endocrine therapy in the adjuvant setting of HGSC. Indeed, this real-world retrospective data analysis contributed to the development of the ongoing ENGOT-ov54/Swiss-GO-2/MATAO trial [NCT04111978] [19].

## 5. Conclusions

ER expression is highly prevalent in HGSC. In the adjuvant setting, letrozole, whether used alone or in combination with standard targeted therapies, demonstrated a low toxicity profile with a low discontinuation rate. Future prospective clinical trials are needed to identify the subgroup of patients with HGSC who derive the greatest benefit from endocrine maintenance therapy. 

## Figures and Tables

**Table 1:** Comparison of the demographic and clinicopathologic characteristics between patients with early maintenance therapy with letrozole and those with no letrozole therapy in high-grade serous ovarian cancer.

Characteristics	All Patients (*n* = 102) *n* (%)	No Letrozole (*n* = 38) *n* (%)	Letrozole (*n* = 64) *n* (%)
Age at diagnosis (years)			
Median	67.0	67.0	67.5
Range	22.0–92.0	50.0–86.0	22.0–92.0
FIGO stage			
I	5 (4.90%)	3 (7.89%)	2 (3.12%)
II	3 (2.94%)	1 (2.63%)	2 (3.12%)
III	60 (58.8%)	23 (60.5%)	37 (57.8%)
IV	34 (33.3%)	11 (28.9%)	23 (35.9%)
Primary site			
Ovary	62 (60.8%)	25 (65.8%)	37 (57.8%)
Fallopian tube	34 (33.3%)	11 (28.9%)	23 (35.9%)
Peritoneum	6 (5.88%)	2 (5.26%)	4 (6.25%)
ER (%)			
Median	70.0	60.0	77.5
Range	1.00–100	1.00–100	1.00–100
*BRCA* status			
No mutation	56 (55.4%)	18 (47.4%)	38 (60.3%)
*BRCA1* mutation	11 (10.9%)	1 (2.63%)	10 (15.9%)
*BRCA2* mutation	11 (10.9%)	3 (7.89%)	8 (12.7%)
No testing	23 (22.8%)	16 (42.1%)	7 (11.1%)
Residual disease status			
R0	50 (52.6%)	23 (65.7%)	27 (45.0%)
R	45 (47.4%)	12 (34.3%)	33 (55.0%)
Neoadjuvant chemotherapy			
Yes	30 (29.4%)	13 (34.2%)	17 (26.6%)
No	72 (70.6%)	25 (65.8%)	47 (73.4%)
Platinum resistance			
Yes	8 (7.8%)	4 (10.5%)	4 (6.3%)
No	94 (92.2%)	34 (89.5%)	60 (93.7%)
Bevacizumab maintenance therapy			
Yes	54 (54.0%)	16 (43.2%)	38 (60.3%)
No	46 (46.0%)	21 (56.8%)	25 (39.7%)
PARP inhibitor maintenance therapy			
Yes	39 (39%)	11 (28.9%)	28 (45.2%)
No	61 (61.0%)	27 (71.1%)	34 (54.8%)

## Data Availability

The datasets that were used and/or analyzed during the current study are available from the corresponding author on reasonable request.

## Supplementary Materials

The following supporting information can be downloaded at: https://www.mdpi.com/article/10.3390/cancers17081301/s1, Table S1: Results of the Kaplan–Meier analysis of progression-free survival of patients with early letrozole maintenance therapy compared to no letrozole maintenance therapy in high-grade serous ovarian cancer with residual disease after primary cytoreductive surgery; Table S2: Results of the Kaplan–Meier analysis of overall survival of patients with early letrozole maintenance therapy compared to no letrozole maintenance therapy in high-grade serous ovarian cancer with residual disease after primary cytoreductive surgery.
