# Supplementary material for: Endocrine Maintenance Therapy in High-Grade Serous Ovarian Cancer: A Retrospective Off-Label Real-World Cohort Study
Source: Cancers (Basel). 2025 Apr 12;17(8):1301. doi: 10.3390/cancers17081301 (PMC12025638; doi:10.3390/cancers17081301)
Supplement: Supplementary file 1 [file cancers-17-01301-s001.zip › cancers-3547735-supplementary.pdf]

Supplementary Tables

Supplementary Table S1. Results of the Kaplan–Meier analysis of progression-free survival of patients with early letrozole maintenance therapy compared to no letrozole maintenance therapy in high-grade serous ovarian cancer with residual disease after primary cytoreductive surgery.

Factor	number	median PFS (months)	0.95LCL	0.95UCL	*P*-value
No Letrozole	9	35.08	4.29	NA	
Letrozole	32	14.33	11.48	20.56	0.17

PFS=Progression-free survival, LCL=Lower Confidence Limit, UCL=Upper Confidence Limit.

Supplementary Table S2. Results of the Kaplan–Meier analysis of overall survival of patients with early letrozole maintenance therapy compared to no letrozole maintenance therapy in high-grade serous ovarian cancer with residual disease after primary cytoreductive surgery.

Factor	number	median OS (months)	0.95LCL	0.95UCL	*P*-value
No Letrozole	9	21.97	19.31	NA	
Letrozole	32	40.59	21.64	NA	0.65

OS=Overall survival, LCL=Lower Confidence Limit, UCL=Upper Confidence Limit.
